# Detecting Nuclear Materials in Urban Environments Using Mobile Sensor Networks

**DOI:** 10.3390/s21062196

**Published:** 2021-03-21

**Authors:** Robert R. Flanagan, Logan J. Brandt, Andrew G. Osborne, Mark R. Deinert

**Affiliations:** 1 Nuclear Science and Engineering, The Colorado School of Mines, Golden, CO 80401, USA; 2 United States Air Force Academy, Colorado Springs, Air Force Academy, CO 80840, USA; 3 Payne Institute for Public Policy, The Colorado School of Mines, Golden, CO 80401, USA

**Keywords:** distributed sensor, radiation detection

## Abstract

Radiation detectors installed at major ports of entry are a key component of the overall strategy to protect countries from nuclear terrorism. While the goal of deploying these systems is to intercept special nuclear material as it enters the country, no detector system is foolproof. Mobile, distributed sensors have been proposed to detect nuclear materials in transit should portal monitors fail to prevent their entry in the first place. In large metropolitan areas, a mobile distributed sensor network could be deployed using vehicle platforms such as taxis, Ubers, and Lyfts, which are already connected to communications infrastructure. However, performance and coverage that could be achieved using a network of sensors mounted on commercial passenger vehicles has not been established. Here, we evaluate how a mobile sensor network could perform in New York City using a combination of radiation transport and geographic information systems. The geographic information system is used in conjunction with OpenStreetMap data to isolate roads and construct a grid over the streets. Vehicle paths are built using pickup and drop off data from Uber, and from the New York State Department of Transportation. The results show that the time to first detection increases with source velocity, decreases with the number of mobile detectors, and reaches a plateau that depends on the strength of the source.

## 1. Introduction

A major concern with the deployment of nuclear power is the potential diversion of nuclear materials for acts of terrorism [1,2]. A fission weapon detonated in a dense population center such as New York City would produce significant casualties as could a dirty bomb and both would cause major disruption. Several radiation detection systems, however, act as a first line defense for the United States. These include radiation detectors at airports, shipyards, commercial ports of entry [3,4], and roadway border crossings [3,4,5]. However, if these systems fail, or if non-official points of entry are used, special nuclear material could be smuggled into the country. Once inside, highways and streets can be used to transport the material to nearly any destination in the United States.

The ease with which passengers and cargo can be moved across the United States presents a unique problem to protecting a city from nuclear terrorism. Distributed detectors could be used here [6,7]. One option is to maintain a stationary detection grid with placement of detectors along entry points to the city. However, smart placement of those systems would be nontrivial [8,9], and although cities like Manhattan have a limited number of access routes such as bridges and tunnels, not all large cities have geographical features that simplify optimal deployment of detector systems.

Another approach to securing a city from nuclear terrorism would be a mobile radiation detection network [10]. The effectiveness of a mobile detector fleet for locating a stationary source has been investigated, where detector vehicles followed fixed or random paths [11]. Advanced machine-learning algorithms have been shown to improve source characterization with multiple sources using mobile detectors [12]. Other studies [13] have focused on detecting mobile sources carried by individuals using stationary detectors, and suggested that it might be possible to use mobile detectors attached to police patrol cars as well. The use of distributed sensor networks to detect stationary sources at highly populated events, such as large sporting events, was investigated in [14]. Taxis, limousines, and ride-share services could provide an ideal platform for detector systems as these vehicles already possess power and communications infrastructure and are ubiquitous in most U.S. cities. However, the effect of source strength, route unpredictability, traffic speed, and detector density remain poorly characterized. In the present work, we evaluate the effect of these variables on a system of mobile sensors mounted on Uber vehicles, to detect a moving radioactive source in Manhattan, NY. The routes taken by the mobile detectors are estimated using historic Uber trip data combined with a route-finding algorithm. Geospatial data on buildings in Manhattan are combined with a simple Green’s function to model radiation transport. The effectiveness of the mobile sensor network is shown as a function of source strength, speed, and the number of detector vehicles.

## 2. Methods

We consider a mobile radioactive source and mobile radiation detectors mounted on Uber vehicles moving through streets in Manhattan, NY. Building geometries in shapefile format were obtained from OpenStreetMap [15]. A limited set of Uber pickup and drop-off zones, and time stamps, are available through the New York Taxi and Limousine Commission [16]. Locations within pickup and drop-off zones were randomly sampled and routes were computed by combining the pickup and drop-off locations and time stamps with the route-finding algorithm provided by the pyroute3 library [17]. The source routes were also determined using pyroute3 and by randomly choosing an origin near the South Manhattan coast and Madison Square Gardens as a destination. The source and mobile detector routes were discretized into equally spaced time indexes with a ∆t of 2 s. The detector routes moved at a constant vehicle speed, which was computed using the total route length and duration. The source route incorporated a 10% random stop chance for each segment of the trip. The duration of the stops ranged from 1 to 10 s to simulate traffic conditions. Geometric data were stored and manipulated using the Shapely [18] python library.

*Radiation transport and detection*. The simulated radioactive source was Co^60^ with a strength of 0.1 and 0.5 Ci, emitting 1.17 and 1.33 MeV gammas. Cobalt 60 was chosen because it is a common nuclear material of concern for dirty bombs [19,20,21]. In the simulations, the source is shielded by 10 cm of lead. Additional shielding from the delivery vehicle itself was assumed to be 0.66 m of air and 1 cm of steel. The gamma flux from the source at a given distance was approximated using a point source Greens function, which accounts for both the inverse square law and attenuation:

(1) $$F\left(r\right)=\overset{M}{\underset{m}{\sum }}\frac{Se^{-r_{m}\mu_{m}}}{4\pi r_{m}^{2}}*D_{A}*D_{E}$$

Here, *F*(*r*) {counts/s} is the strength of the source at distance *r* {m}, *m* is the material the radiation is passing through *µ_m_* {m^-1^} is the linear attenuation coefficient for gammas in material *m*, *r_m_* is the distance the radiation moves through material *m*, *S* {counts/s} is the strength of the source after being attenuated by the shielding, *D_E_* is the detector efficiency, and *D_A_* is the detector area. The detector is assumed to be a scintillating detector used to count gross counts. Gross counts are also used for the background rate.

The maximum range at which the source can be detected is assumed to be the distance at which the source drops to be indistinguishable from the background gamma radiation count rate for Manhattan, ~83 counts/s, given by the RadNet [22]. The ranges before the source strengths dropped to the background were determined to be 24.43 m for the 0.5 Ci and 5.12 m for the 0.1 Ci sources. For this work, a detection was recorded when the count rate at a detector exceeded the background count rate by three standard deviations. Counts were integrated using the count rate detected at a time step and then multiplying that value by the length of the time step (2 s). The probability of false alarm for such a detection threshold is less than 0.5%. This standard deviation was calculated assuming the background radiation fits a Poisson distribution; meaning the standard deviation is equal to the square root of the mean. The background count rate was assumed to be constant throughout the city for this work. This is not fully realistic, as background rates vary both spatially and temporally in urban environments [23,24]. However, a fleet of mobile detectors could map out a spatial and temporal background that could be used for future research [25].

*Source route generation*. Generation of the source routes was done by randomly sampling points along the southern tip of Manhattan. For each point sampled, a route was built between the initial starting point and the destination of Madison Square Gardens. Routes were built, and routes from 100 randomly chosen starting points were generated.

*Detector route data*. Data for Uber pickup and drop-off zones for New York City for 2016–2020 were taken from the New York Taxi and Limousine Commission [16]. These data provide ~70,000 pickup and drop-off zone pairs per day, with ~20 h of coverage. A sample of these data for 13 December 13 2019, from 12:00 to 12:20 p.m., was chosen at random, and provided 3500 pickup and drop-off zone pairs. The route-finding algorithm provided by the pyroute3 library [17] was used with these to determine the shortest route between the two locations, and this was assumed to be the one used by the simulated mobile detectors.

*Source and detector routes*. Once the source and detector routes were constructed and broken into points with timestamps, these points are used to create a Sort Tile Recursive Tree (STRtree) using the Shapely python library. The STRtree allows for quick querying of information about the points in relation to each other. For each point in the source path, the STRtree is queried to determine which detectors are in range of the source at the given timestamp. This range is determined by calculating the maximum distance that the source could be detected given attenuation from Equation (1). Again, the detection limit was set to be three standard deviations above background. For each detector in range, the distance from the source to the detector was calculated and the number of counts per second the detector measures recorded. Buildings intersecting the path between source and detector were assumed to absorb all radiation. In this work, radiation transport through traffic other than the source and detector vehicles was ignored. 

*Source velocities*. Three speeds were chosen to represent the movement of the source relative to data available from the NYC Department of Transportation for the average speed of bus traffic (3.1 m/s) in Manhattan [26]: 2 m/s was used to represent a vehicle source moving through heavy traffic; 10 m/s was chosen to represent fast moving traffic; and 15 m/s was chosen to represent a motorcycle moving through city streets.

## 3. Results

Considerable work has been done to flag radioactive materials as they pass through ports of entry, or major transit points. Finding mobile nuclear materials in an urban environment is a much more difficult problem and requires non-traditional deployment of detectors [27,28]. Mobile detectors can play an important role here in decreasing the risks posed by radiological sources by providing real-time radiation monitoring.

**Figure 1:**
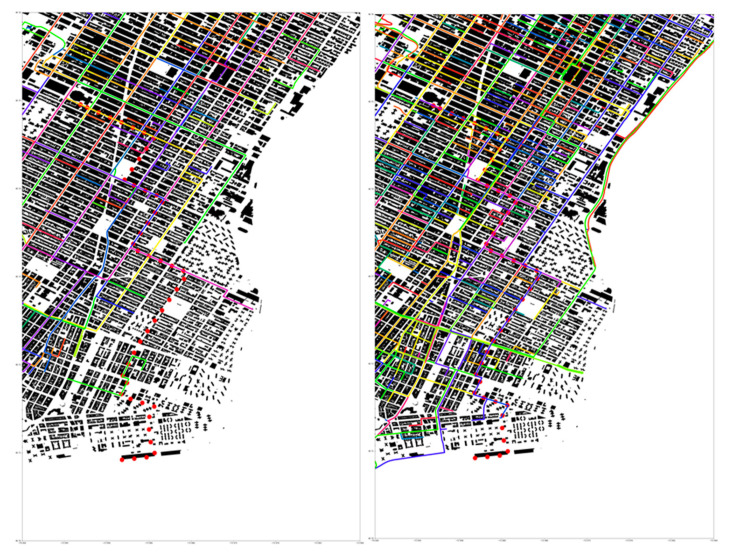
Visualization of 100 and 500 Uber routes on 3 December 2018. (**Left**) 100 Uber tracks and (**right**) 500 tracks sampled from 3500 routes that occurred between 3:00 p.m. and 3:20 p.m. on this date. The solid lines represent the Uber traffic. The red dotted line represents the source path. All journeys begin and end inside the 20-min duration. Journeys that start or finish outside the duration were excluded. This figure highlights the areas of good coverage and shows weaker coverage toward the coasts.

Figure 1 shows routes generated for 3 December 2018 from 3:00 pm until 3:20 pm. While a total of 3500 Uber journeys were made in that period, the number of routes sampled for performing the simulation was varied to evaluate the detector network’s performance with increasing numbers of detector vehicles. Figure 1 shows Uber tracks for 100 (left) and 500 (right) sampled routes. The solid-colored lines represent the Uber tracks, and the red dotted line is one of the sampled source paths. Buildings are shown as solid black objects, and the white space represents roads, alleyways, parks, parking lots, and other space unoccupied by a building. Although Figure 1 shows that increasing the number of Uber routes sampled increases the cumulative coverage of the city this does not guarantee that this source and a given detector are coincident.

**Figure 2:**
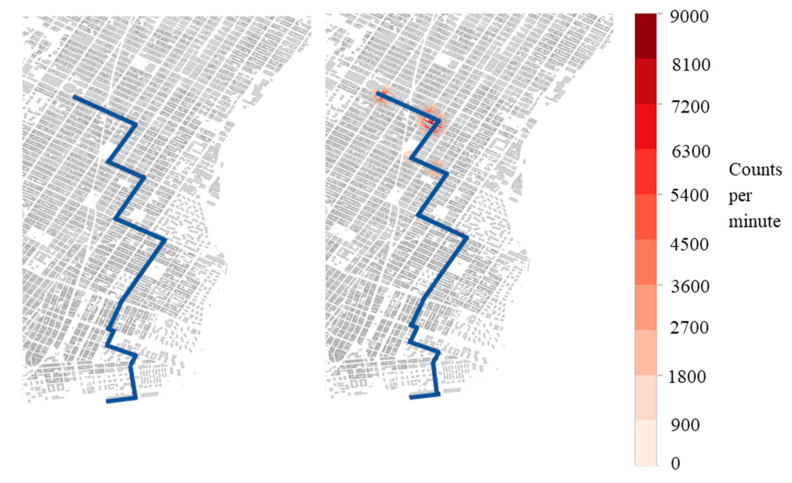
Detection heat map. The map shows the counts per minute above background detected for a single route for a 200-vehicle mobile detector array of a 0.1 Ci Co-60 (**left**—case 2) and a 0.5 Ci Co-60 (**right**—case 5) source moving at 10 m/s.

Table 1 summarizes results from 12 simulated cases with source speeds that varied from 2 to 15 m/s, strengths of either 0.1 or 0.5 Ci, and with either 200 or 400 mobile detectors deployed. In each case, the number of times a source was detected during its transit was recorded. The results in the table show that by increasing the speed, the detection rate decreases. Additionally, as the detector count increased, the number of detections increased. Both observations are in line with the expected outcome. The left image in Figure 2 shows the heat map for Case 2 (10 m/s) for a specific source route. A small number of counts is detected in the region of the last turn. The low detection in these three cases stems from a weaker source, resulting in a smaller detection radius, as well as low sensor density.

**Figure 3:**
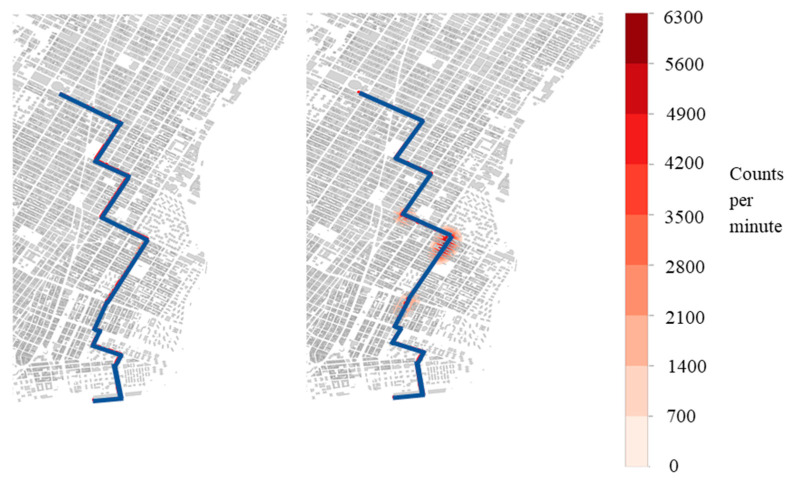
Detection heat map. Maps show the counts per minute above background detected for a single route for a 400-vehicle mobile detector array of a 0.1 Ci Co-60 (**left**—case 7) and a 0.5 Ci Co-60 (**right**—case 10) source moving at 2 m/s.

**Figure 4:**
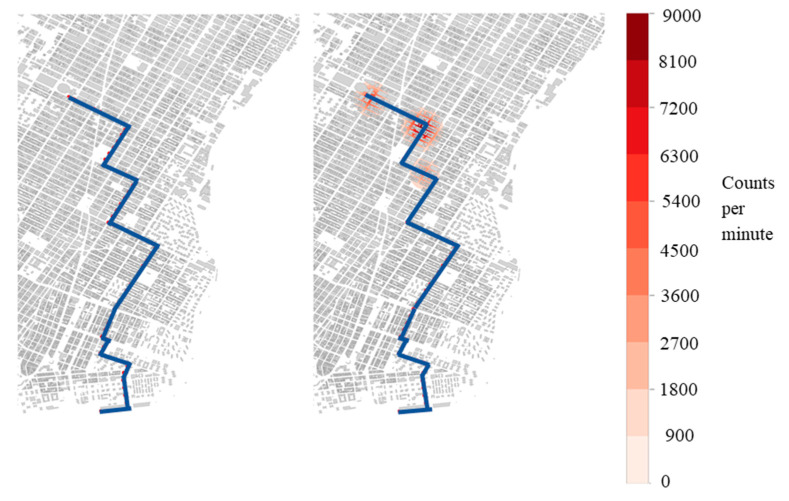
Detection heat maps. Maps show the counts per minute above background detected for a single route from a 400-vehicle mobile detector array of a 0.1 Ci Co-60 (**left**—case 8) and a 0.5 Ci Co-60 (**right**—case 11) source moving at 10 m/s.

Figure 2, Figure 3 and Figure 4 show the detection locations of a sample source route moving through 200 and 400 detectors. These figures show that, for a given route, the behavior of detection is not consistent with the larger sample of the 100 source routes, and is the reasoning for that increased sample size. The discussion of the figures highlights the behavior associated with one specific source route, not the collection of source routes.

Figure 2 shows the count rate that a fleet of 200 detectors following historic Uber routes, during 3 December 2018 from 3:00 p.m. until 3:20 p.m., would measure from a sample 0.1 and 0.5 Ci source moving at 10 m/s as a function of position (Table 1 cases 2 and 5). Count rates shown are summed over all detectors in range and represent a count rate above background. The mobile detectors were only able to detect the signal of the 0.1 Ci source at one location. By contrast, the 0.5 Ci source was detected at three locations along the route, which comes from the increase in detection radius due to the higher source strength.

Figure 3 shows the count rate for 400 detectors and a source speed of 2 m/s (Table 1 Cases 7 and 10). These results again show that higher source strength increases the number of detections with three points of detection for the stronger source. However, the locations at which these occur are different from those found in Figure 2, because the speed of the source causes it to be detected by a different set of mobile detectors.

Figure 4 shows the count rate for 400 detectors and a 10 m/s source speed (Table 1 Cases 8 and 11). These results again show that increased source strength causes increased detections. These two cases can be compared directly to the cases shown in Figure 2. Figure 4 shows that increasing the number of detectors increased the number of detection locations from three to four. The reason is that the higher speed results in the source passing by more mobile detectors and registering more detections as a result.

Cases 4,5,6 were evaluated for 200 mobile sensors and a 0.5 Ci source, with source speed from 2 to 15 m/s. Here we found a similar situation to the cases 1,2,3. The 2m/s and 15m/s cases (4, 6) showed no detection of the source. However, there is an improvement in the detection between cases 2 and 5 due to a factor of five increase in source strength, from 0.1 to 0.5Ci. The integrated count value in Case 5 is 2.34 × 105, which is more than a factor of five larger than Case 2 (3.27 × 104 counts). This is because a stronger source increases the minimum detection radius, resulting in more detectors being in range to measure a signal. 

In Cases 7,8,9, 400 detectors were simulated with a 0.1 Ci source with source speed varying from 2 to 15 m/s. For this source route, the 10 m/s source velocity sources have the highest detection metrics in most scenarios, but this trend is not true when all sampled routes are considered. As seen in Table 1, increasing source speed decreases the number of total detections across all source routes. A faster speed means the amount of time required to get to the destination is reduced and therefore the amount of time for detections is also reduced. The reason for this discrepancy is that the location of detectors and sources are constantly moving. Therefore, coincidence of these locations drives the detection rates for an individual source route. 

**Figure 5:**
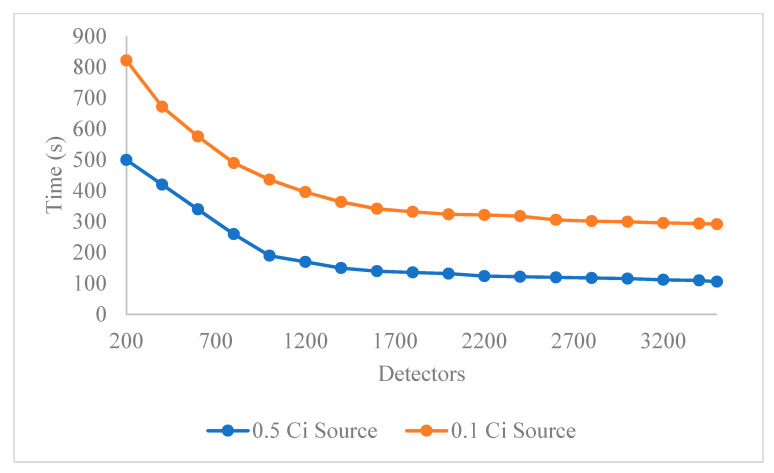
The average time of first detection from the start of the source routes. This calculation was done for sources moving at 10 m/s.

Figure 5 shows the average time of first detection for a source moving at 10 m/s for a varying number of mobile detectors. For the 0.5 Ci source this figure shows that increasing the number of detectors, decreases the average time of first detection, but only to a point. The initial portion of each source trip moves through a region where ride shares have a low density. The marginal gains beyond 1000 detectors likely reflects the source being detected very quickly once it enters a region of high ride share activity, with only marginal gains with subsequent increases in detector number. For the 0.1 Ci source increasing the number of detectors again decreases the time of first detection with the same basic profile as seen in the 0.5 Ci case. For the weaker source strength, the reduction in average detection time appears to even out around 1700 detectors. However, due to the lower source strength, the detection time remains higher than for the 0.1 Ci case over the 3400 routes available for this work.

The are several limitations with the results presented here. The mobile detectors simulated here operate independently of one another. This means that the detection probability rests solely with the individual sensors, and it is not possible to integrate signals that operate near background to get a better indication of a mobile source. The effect of attenuation from traffic and buildings was also not considered. The former is considered to play a minor role, and the latter are radiologically opaque. Buildings (especially at their corners) would offer less attenuation and traffic more. High traffic would increase the effective attenuation coefficient by potentially placing more vehicles in the way between the source and the detector. In both cases, accurate models for attenuation need to be developed. Another limitation of this work is the route-finding software. Due to the resolution of the software, the placement of the vehicles at each time step could overlap (narrow streets with two lanes may not resolve cars in each lane, but rather place them in the center of the two lanes). The effect of this limitation would be to add uncertainty to the distance between two vehicles, but only if the vehicles were on a narrow street. Additionally, while this work used realistic data from ride shares in New York City, only pickup and drop off zones were provided with the ride share data. Exact locations needed to be selected within those zones, resulting in recreated routes similar to the original routes, but likely not the exact same. This limitation was deemed acceptable for this work because it is likely that there is an element of randomness within the ride share travel from day to day.

This work does not explore the economic cost of outfitting the ride share vehicles with detectors of the design indicated in this work. Additionally, economic analysis was not done to understand the relationship between money spent on detectors and the likelihood of detection. Future work should explore these areas to understand the monetary costs associated with this method of detection.

This work highlights the effectiveness of a mobile detector system, but a stationary system of detectors could also be used to detect radiological threats. Of interest here would be the detector density compared to the effectiveness of the detectors and the costs associated with that density. Additional work could also be done to show the benefits of coupling the two systems. The economics of the two systems, or the couple system, would be valuable information for determining detection strategies.

## 4. Conclusions

This work shows that mobile sensors on vehicle platforms, without explicitly designated routes such as ride share vehicles, could be used to locate radioactive sources in a city. The results show that increasing the number of detectors decreases the time of first detection. However, the benefit of increasing detectors decreases after 1000 detectors has been reached for a 0.5Ci source and is reached with 1700 detectors for a 0.1Ci one. This asymptotic behavior is likely the result of the ride shares not going into the starting location of the source routes. This may be due to the coast of the city being a less well trafficked area.

Further work should investigate detection probabilities at different times of day to account for varying levels of traffic, which would impact both vehicle speed and vehicle density. Higher fidelity radiation transport models should be investigated to see their impact on the results. While strong gamma emitting 0.5 and 0.1 Ci ^60^Co sources were used in this work, realistic situations could involve much weaker sources, as well as beta and alpha emitters such as ^210^Po and ^90^Sr which would be significantly more difficult to detect. Our method can be extended in a simple way, however, to establish whether detection of these types of emitters is feasible. Incorporating stationary sensors, and detectors of varying types mounted on a range of portable platforms such as Unmanned Aerial Vehicles and hand-held devices could dramatically improve the sensor network’s effectiveness and can be readily implemented into our existing analysis tools [29,30].

## Figures and Tables

**Table 1:** 100 cases were simulated with varying source speed and strength for 200 or 400 detectors. Source paths were identical in each case. Mobile sensors followed 200 or 400 identical routes.

Case Number	Speed of Source (m/s)	Speed of Source (mph)	Strength of Source (Ci)	Number of Mobile Detectors	Detection Occurrences
1	2	4.47	0.1	200	86
2	10	22.37	0.1	200	68
3	15	33.55	0.1	200	62
4	2	4.47	0.5	200	238
5	10	22.37	0.5	200	147
6	15	33.55	0.5	200	119
7	2	4.47	0.1	400	101
8	10	22.37	0.1	400	93
9	15	33.55	0.1	400	72
10	2	4.47	0.5	400	357
11	10	22.37	0.5	400	306
12	15	33.55	0.5	400	154

## Data Availability

All data is publicly available and listed in the references.
